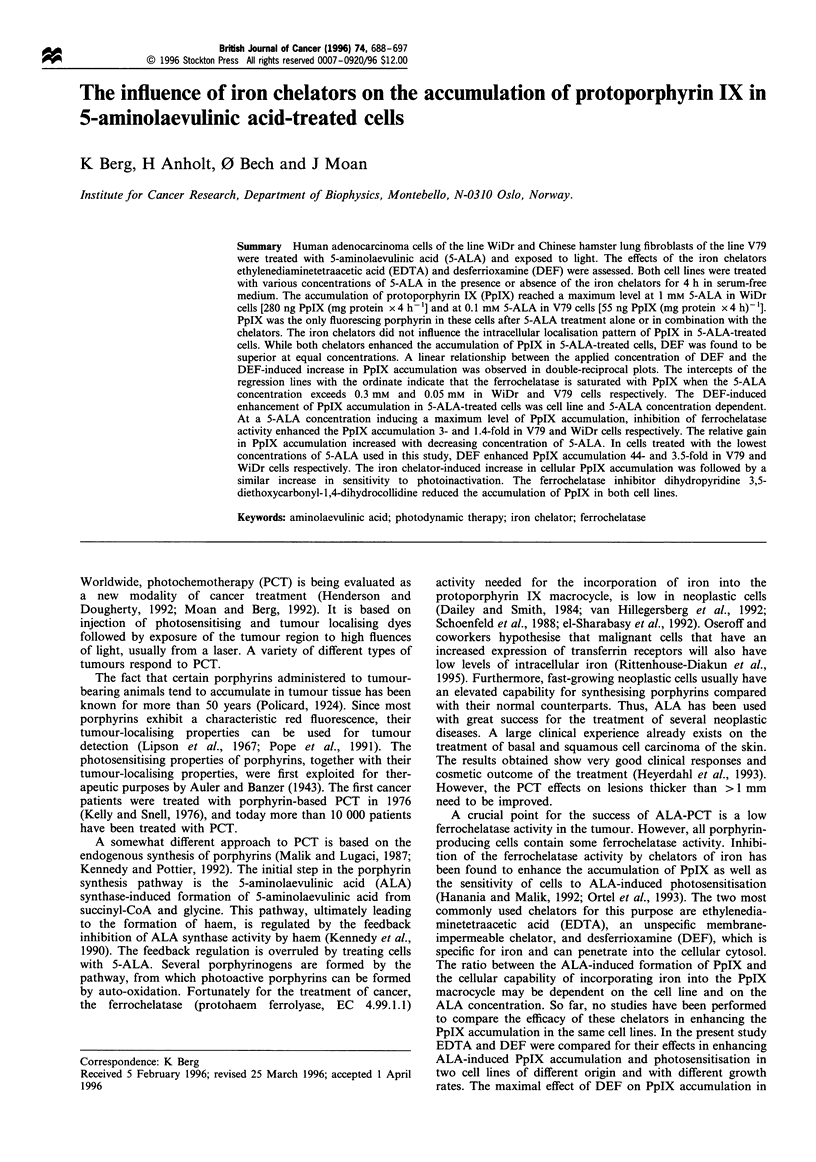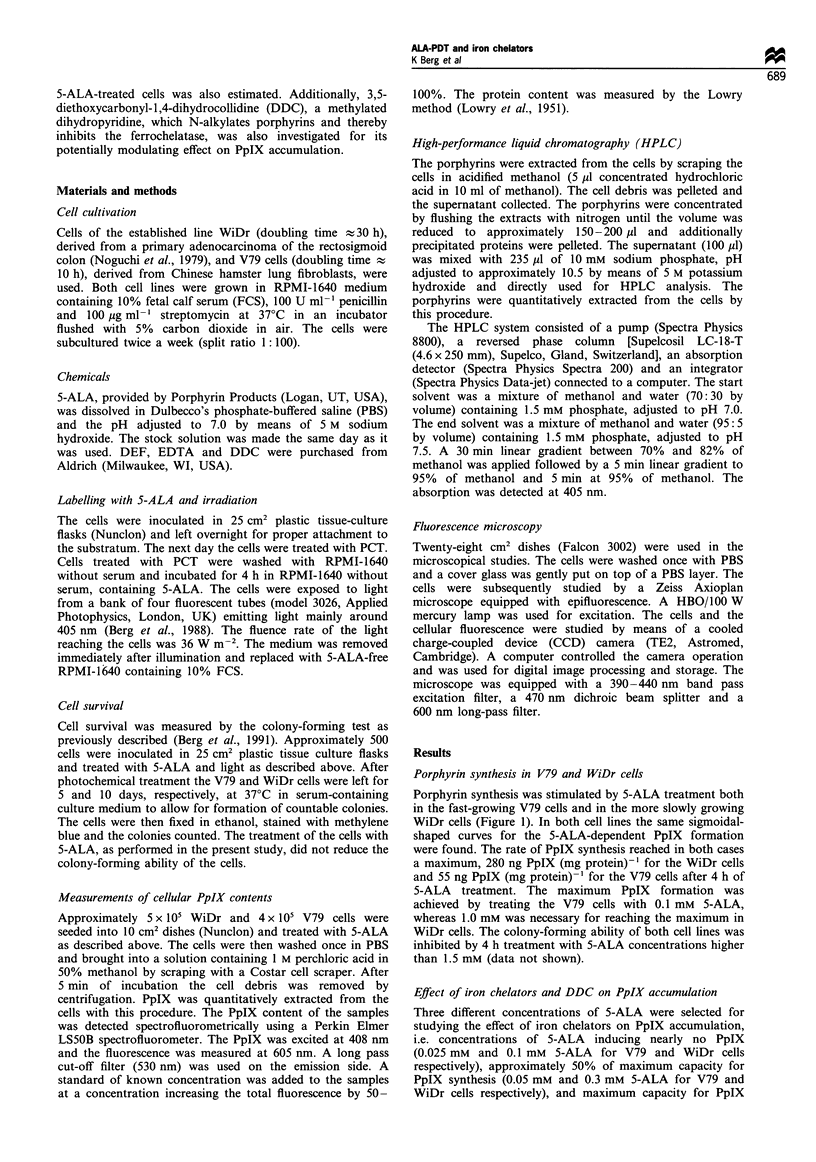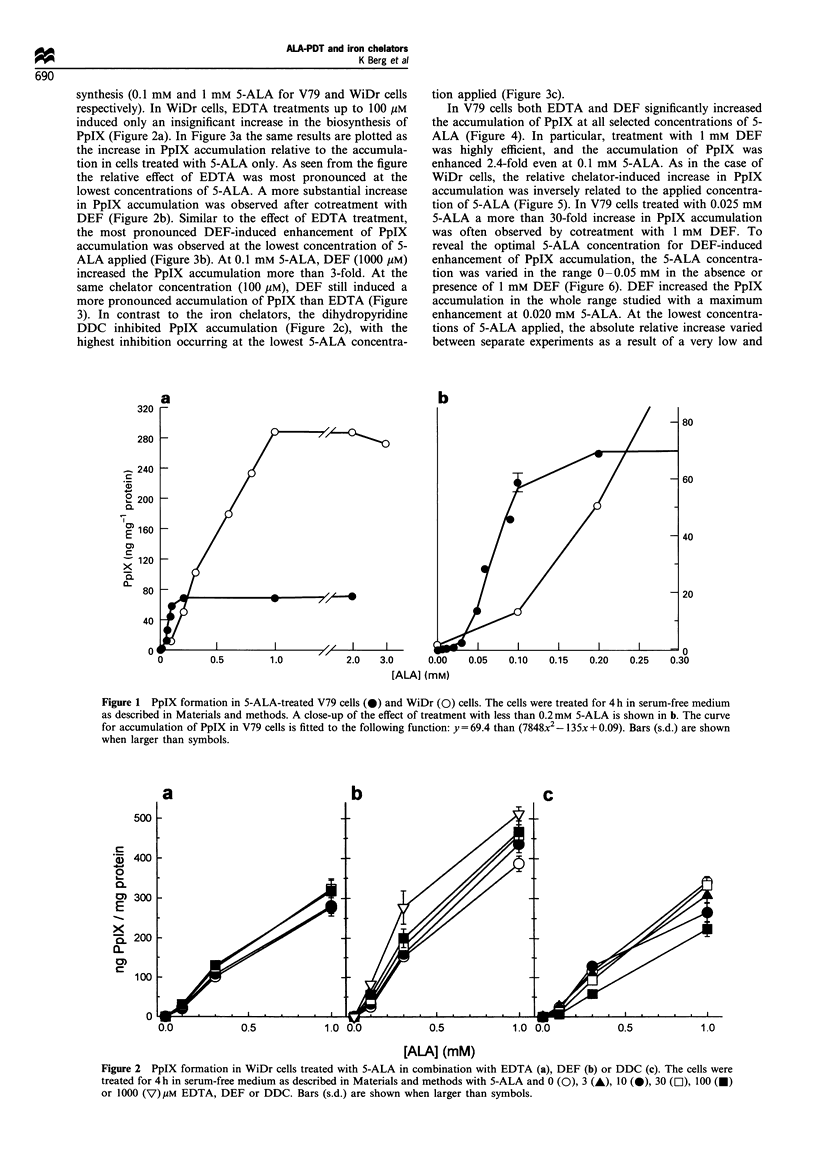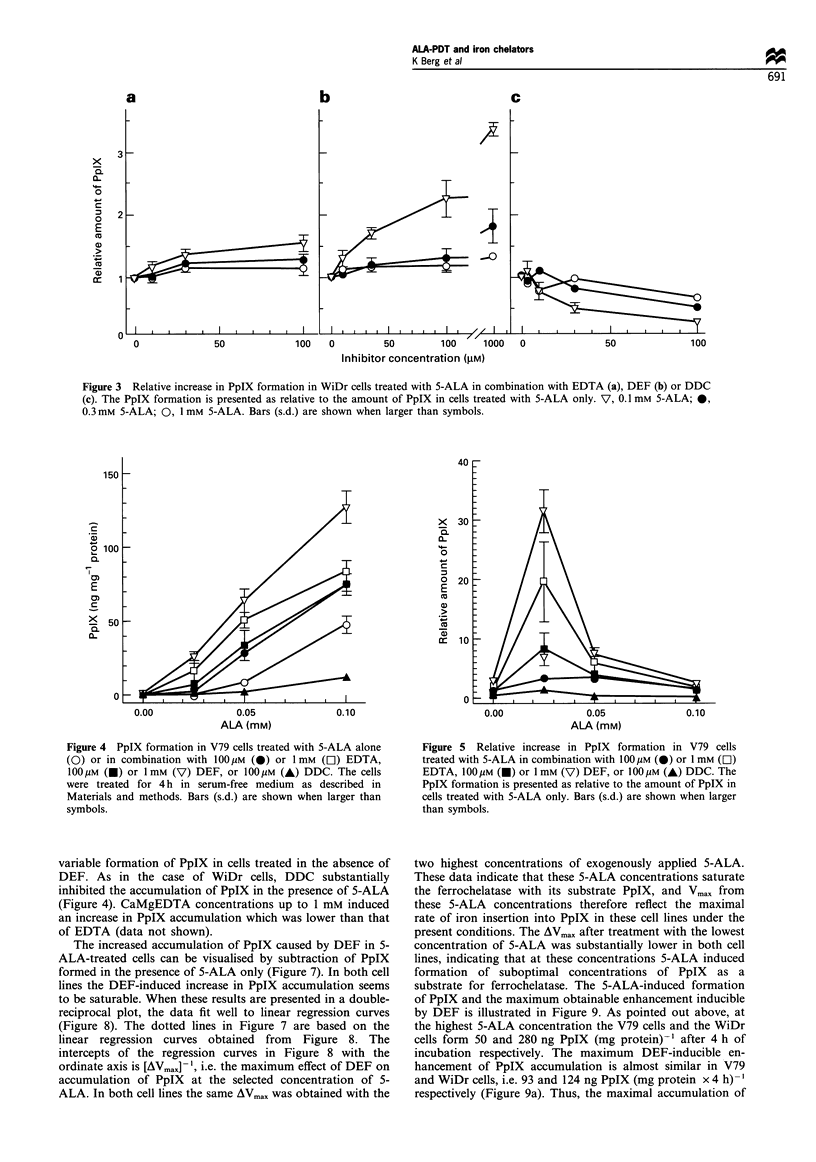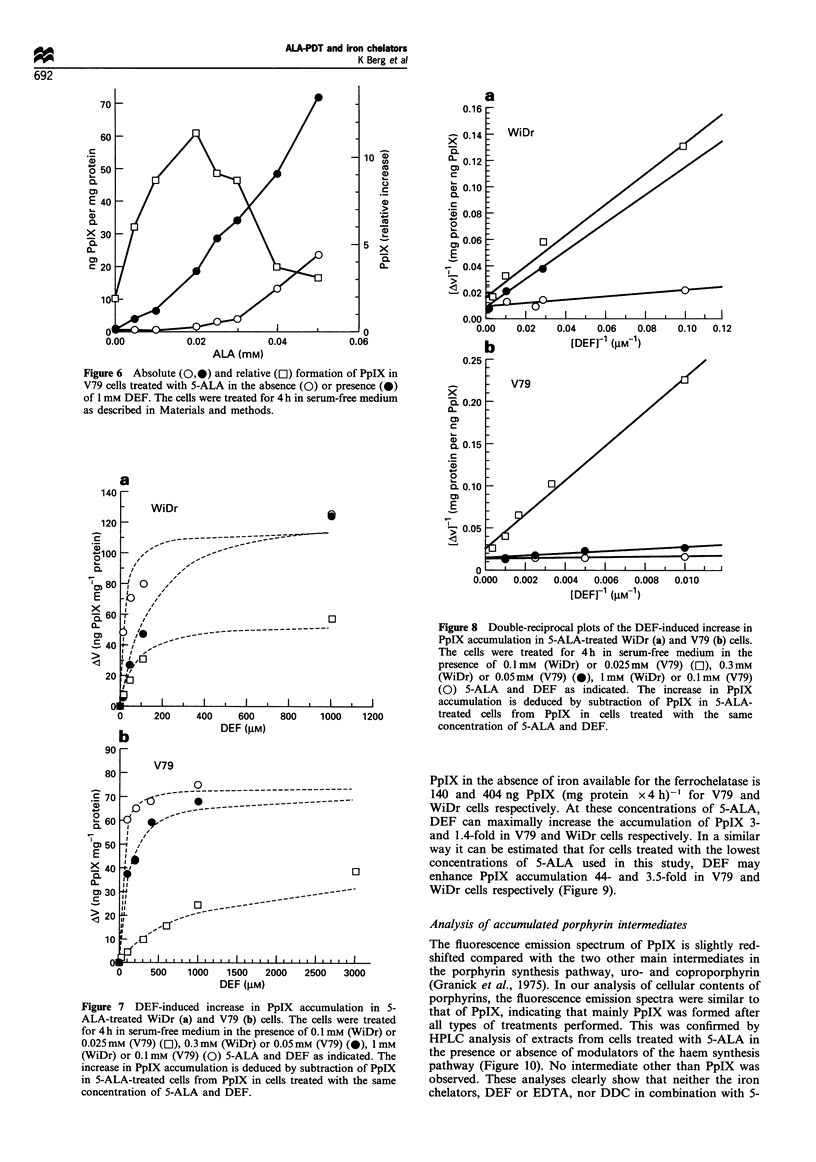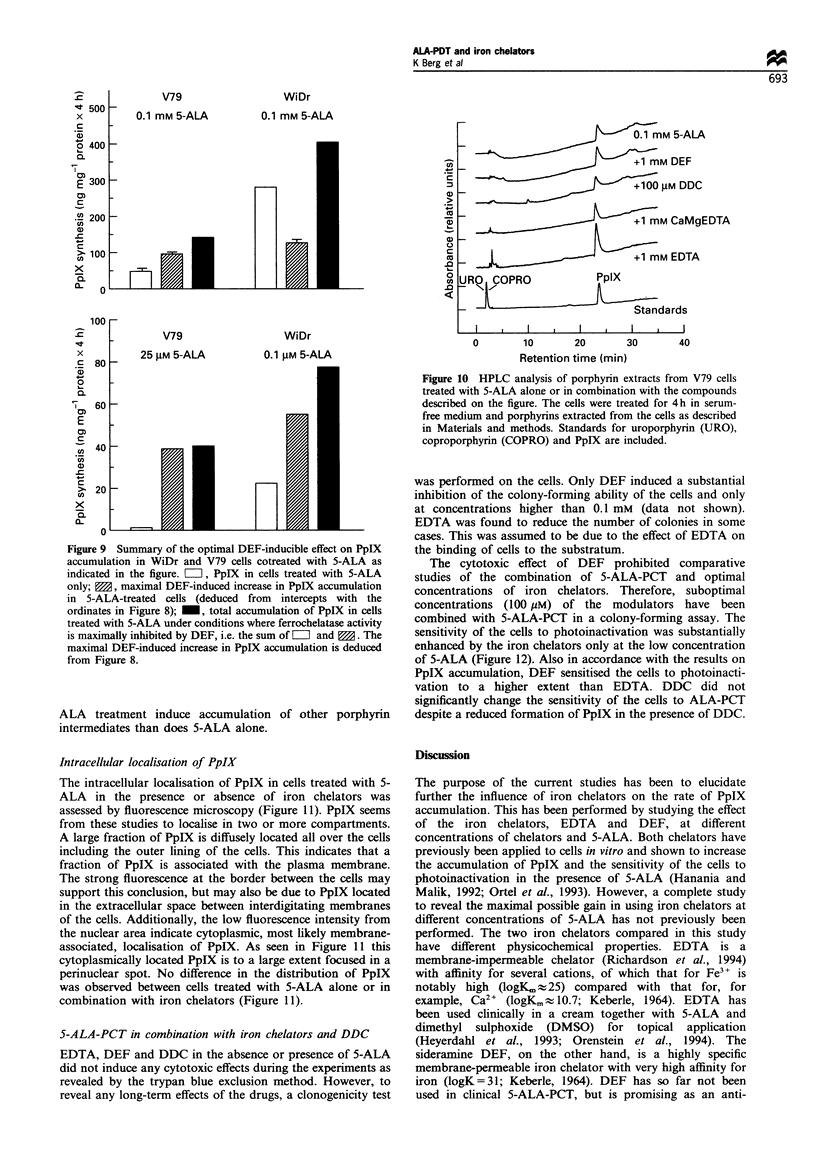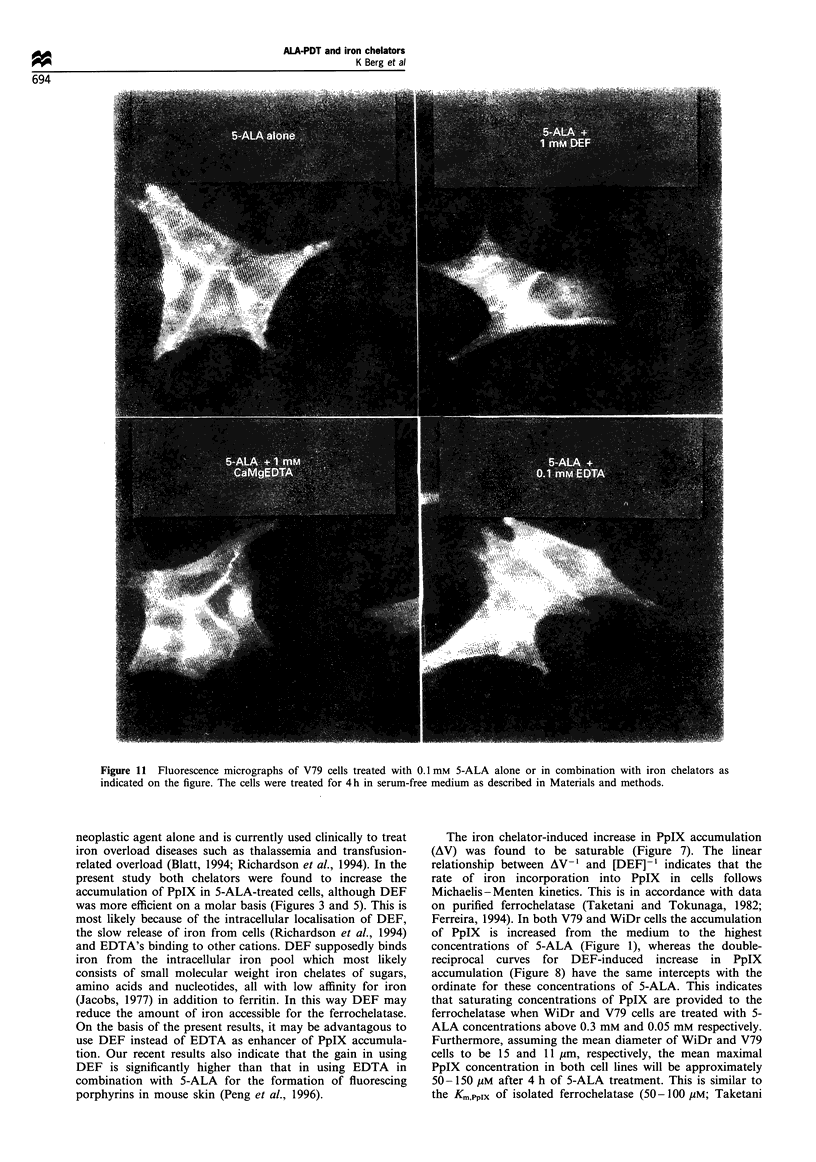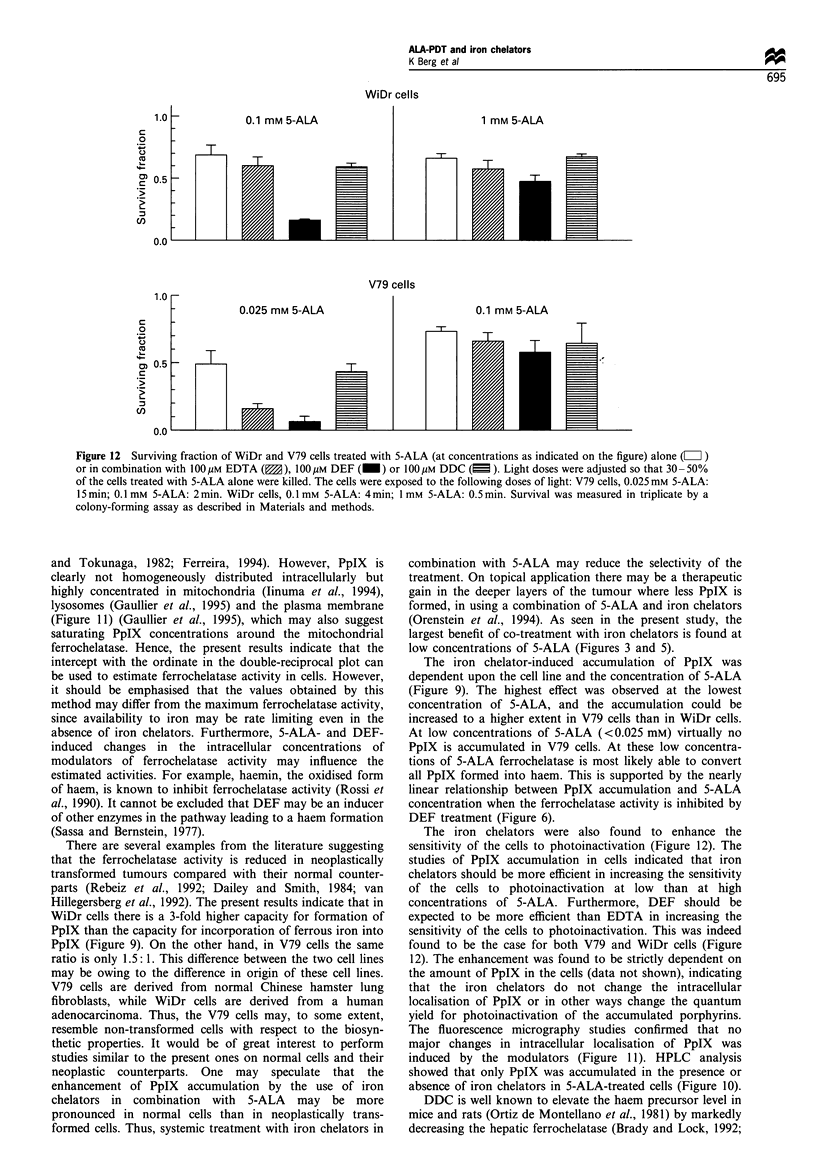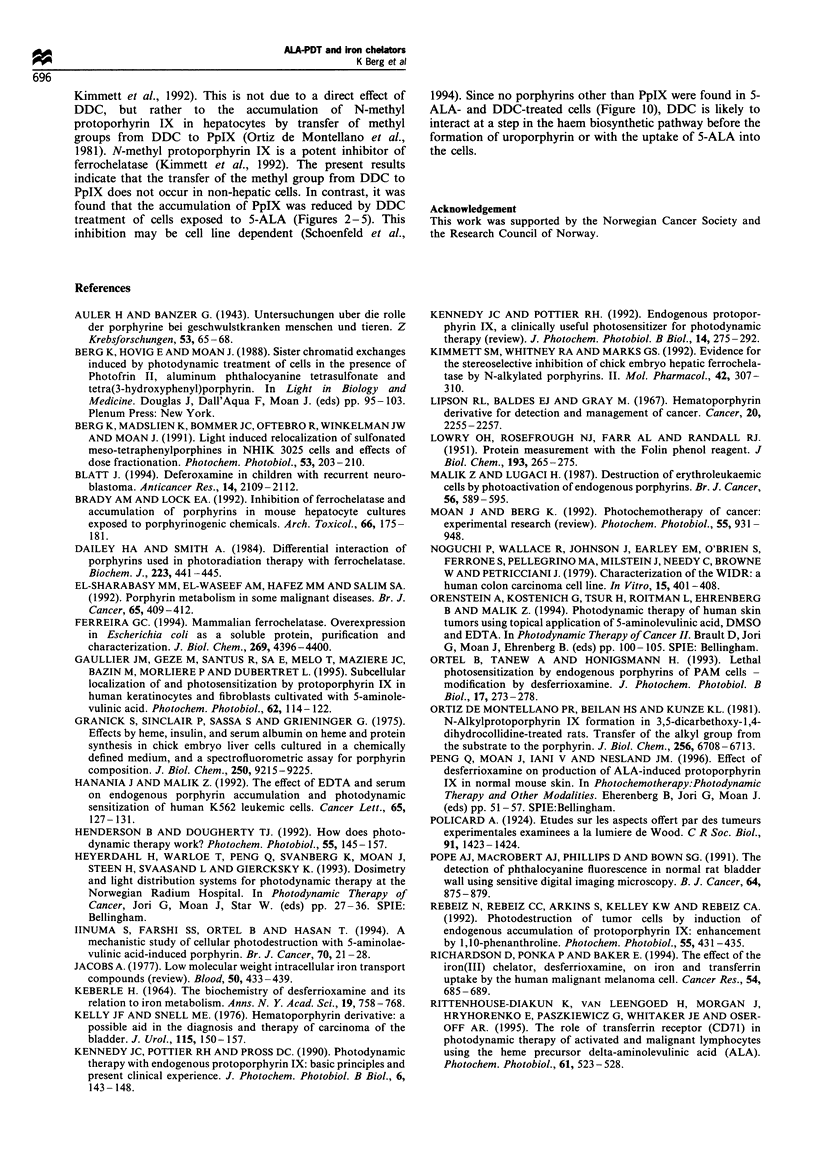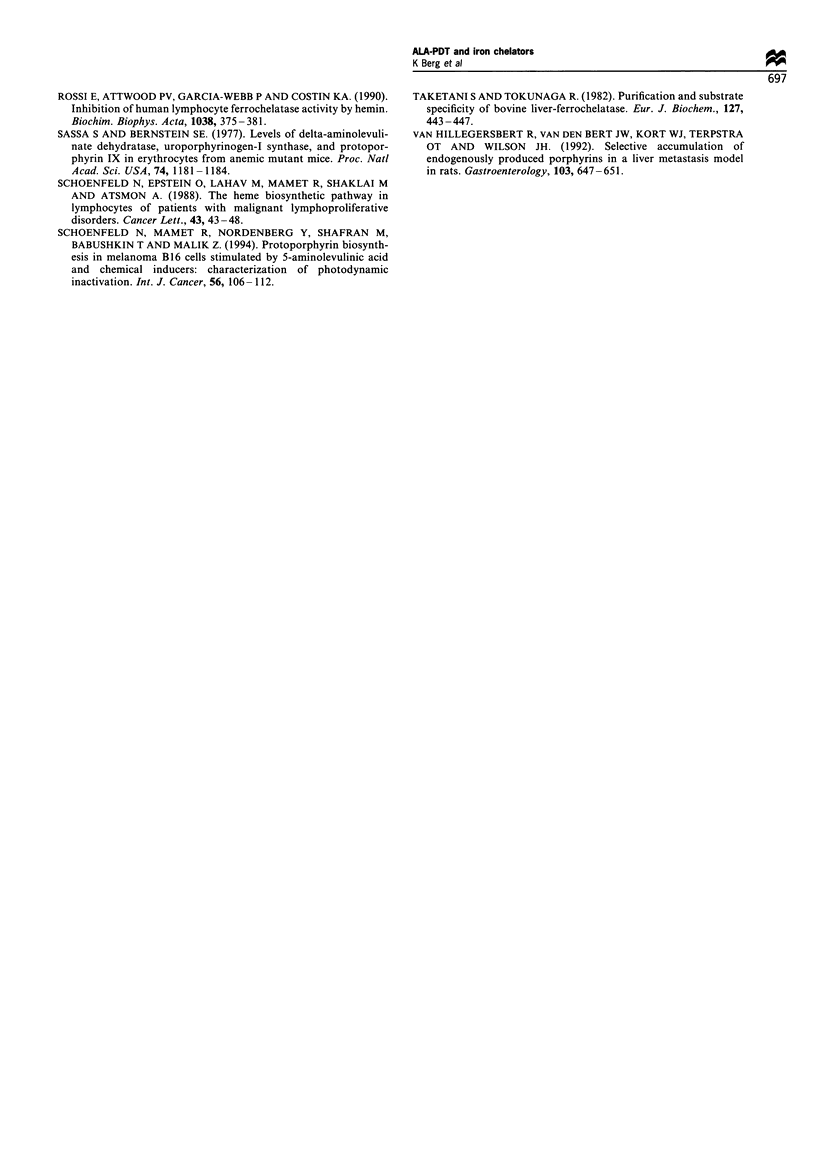# The influence of iron chelators on the accumulation of protoporphyrin IX in 5-aminolaevulinic acid-treated cells.

**DOI:** 10.1038/bjc.1996.423

**Published:** 1996-09

**Authors:** K. Berg, H. Anholt, O. Bech, J. Moan

**Affiliations:** Institute for Cancer Research, Department of Biophysics, Montebello, Norway.

## Abstract

**Images:**


British Journal of Cancer (1996) 74, 688-697
© 1996 Stockton Press All rights reserved 0007-0920/96 $12.00

### The influence of iron chelators on the accumulation of protoporphyrin IX in 5-aminolaevulinic acid-treated cells

K Berg, H Anholt, Ø Bech and J Moan

*Institute for Cancer Research, Department of Biophysics, Montebello, N-0310 Oslo, Norway.*

**Summary** Human adenocarcinoma cells of the line WiDr and Chinese hamster lung fibroblasts of the line V79 were treated with 5-aminolaevulinic acid (5-ALA) and exposed to light. The effects of the iron chelators ethylenediaminetetraacetic acid (EDTA) and desferrioxamine (DEF) were assessed. Both cell lines were treated with various concentrations of 5-ALA in the presence or absence of the iron chelators for 4 h in serum-free medium. The accumulation of protoporphyrin IX (PpIX) reached a maximum level at 1 mM 5-ALA in WiDr cells [280 ng PpIX (mg protein $\times 4$ $h^{-1}$] and at 0.1 mM 5-ALA in V79 cells [55 ng PpIX (mg protein $\times 4$ h$)^{-1}$]. PpIX was the only fluorescing porphyrin in these cells after 5-ALA treatment alone or in combination with the chelators. The iron chelators did not influence the intracellular localisation pattern of PpIX in 5-ALA-treated cells. While both chelators enhanced the accumulation of PpIX in 5-ALA-treated cells, DEF was found to be superior at equal concentrations. A linear relationship between the applied concentration of DEF and the DEF-induced increase in PpIX accumulation was observed in double-reciprocal plots. The intercepts of the regression lines with the ordinate indicate that the ferrochelatase is saturated with PpIX when the 5-ALA concentration exceeds 0.3 mM and 0.05 mM in WiDr and V79 cells respectively. The DEF-induced enhancement of PpIX accumulation in 5-ALA-treated cells was cell line and 5-ALA concentration dependent. At a 5-ALA concentration inducing a maximum level of PpIX accumulation, inhibition of ferrochelatase activity enhanced the PpIX accumulation 3- and 1.4-fold in V79 and WiDr cells respectively. The relative gain in PpIX accumulation increased with decreasing concentration of 5-ALA. In cells treated with the lowest concentrations of 5-ALA used in this study, DEF enhanced PpIX accumulation 44- and 3.5-fold in V79 and WiDr cells respectively. The iron chelator-induced increase in cellular PpIX accumulation was followed by a similar increase in sensitivity to photoinactivation. The ferrochelatase inhibitor dihydropyridine 3,5-diethoxycarbonyl-1,4-dihydrocollidine reduced the accumulation of PpIX in both cell lines.

**Keywords:** aminolaevulinic acid; photodynamic therapy; iron chelator; ferrochelatase

Worldwide, photochemotherapy (PCT) is being evaluated as a new modality of cancer treatment (Henderson and Dougherty, 1992; Moan and Berg, 1992). It is based on injection of photosensitising and tumour localising dyes followed by exposure of the tumour region to high fluences of light, usually from a laser. A variety of different types of tumours respond to PCT.

The fact that certain porphyrins administered to tumour-bearing animals tend to accumulate in tumour tissue has been known for more than 50 years (Policard, 1924). Since most porphyrins exhibit a characteristic red fluorescence, their tumour-localising properties can be used for tumour detection (Lipson *et al.*, 1967; Pope *et al.*, 1991). The photosensitising properties of porphyrins, together with their tumour-localising properties, were first exploited for therapeutic purposes by Auler and Banzer (1943). The first cancer patients were treated with porphyrin-based PCT in 1976 (Kelly and Snell, 1976), and today more than 10 000 patients have been treated with PCT.

A somewhat different approach to PCT is based on the endogenous synthesis of porphyrins (Malik and Lugaci, 1987; Kennedy and Pottier, 1992). The initial step in the porphyrin synthesis pathway is the 5-aminolaevulinic acid (ALA) synthase-induced formation of 5-aminolaevulinic acid from succinyl-CoA and glycine. This pathway, ultimately leading to the formation of haem, is regulated by the feedback inhibition of ALA synthase activity by haem (Kennedy *et al.*, 1990). The feedback regulation is overruled by treating cells with 5-ALA. Several porphyrinogens are formed by the pathway, from which photoactive porphyrins can be formed by auto-oxidation. Fortunately for the treatment of cancer, the ferrochelatase (protohaem ferrolyase, EC 4.99.1.1) activity needed for the incorporation of iron into the protoporphyrin IX macrocycle, is low in neoplastic cells (Dailey and Smith, 1984; van Hillegersberg *et al.*, 1992; Schoenfeld *et al.*, 1988; el-Sharabasy *et al.*, 1992). Oseroff and coworkers hypothesise that malignant cells that have an increased expression of transferrin receptors will also have low levels of intracellular iron (Rittenhouse-Diakun *et al.*, 1995). Furthermore, fast-growing neoplastic cells usually have an elevated capability for synthesising porphyrins compared with their normal counterparts. Thus, ALA has been used with great success for the treatment of several neoplastic diseases. A large clinical experience already exists on the treatment of basal and squamous cell carcinoma of the skin. The results obtained show very good clinical responses and cosmetic outcome of the treatment (Heyerdahl *et al.*, 1993). However, the PCT effects on lesions thicker than >1 mm need to be improved.

A crucial point for the success of ALA-PCT is a low ferrochelatase activity in the tumour. However, all porphyrin-producing cells contain some ferrochelatase activity. Inhibition of the ferrochelatase activity by chelators of iron has been found to enhance the accumulation of PpIX as well as the sensitivity of cells to ALA-induced photosensitisation (Hanania and Malik, 1992; Ortel *et al.*, 1993). The two most commonly used chelators for this purpose are ethylenediaminetetraacetic acid (EDTA), an unspecific membrane-impermeable chelator, and desferrioxamine (DEF), which is specific for iron and can penetrate into the cellular cytosol. The ratio between the ALA-induced formation of PpIX and the cellular capability of incorporating iron into the PpIX macrocycle may be dependent on the cell line and on the ALA concentration. So far, no studies have been performed to compare the efficacy of these chelators in enhancing the PpIX accumulation in the same cell lines. In the present study EDTA and DEF were compared for their effects in enhancing ALA-induced PpIX accumulation and photosensitisation in two cell lines of different origin and with different growth rates. The maximal effect of DEF on PpIX accumulation in

Correspondence: K Berg
Received 5 February 1996; revised 25 March 1996; accepted 1 April 1996

5-ALA-treated cells was also estimated. Additionally, 3,5-diethoxycarbonyl-1,4-dihydrocollidine (DDC), a methylated dihydropyridine, which N-alkylates porphyrins and thereby inhibits the ferrochelatase, was also investigated for its potentially modulating effect on PpIX accumulation.

### Materials and methods

### *Cell cultivation*

Cells of the established line WiDr (doubling time ≈30 h), derived from a primary adenocarcinoma of the rectosigmoid colon (Noguchi *et al.*, 1979), and V79 cells (doubling time ≈ 10 h), derived from Chinese hamster lung fibroblasts, were used. Both cell lines were grown in RPMI-1640 medium containing 10% fetal calf serum (FCS), 100 U $ml^{-1}$ penicillin and 100 $\mu g$ $ml^{-1}$ streptomycin at 37°C in an incubator flushed with 5% carbon dioxide in air. The cells were subcultured twice a week (split ratio 1 : 100).

### *Chemicals*

5-ALA, provided by Porphyrin Products (Logan, UT, USA), was dissolved in Dulbecco's phosphate-buffered saline (PBS) and the pH adjusted to 7.0 by means of 5 M sodium hydroxide. The stock solution was made the same day as it was used. DEF, EDTA and DDC were purchased from Aldrich (Milwaukee, WI, USA).

### *Labelling with 5-ALA and irradiation*

The cells were inoculated in 25 $cm^2$ plastic tissue-culture flasks (Nunclon) and left overnight for proper attachment to the substratum. The next day the cells were treated with PCT. Cells treated with PCT were washed with RPMI-1640 without serum and incubated for 4 h in RPMI-1640 without serum, containing 5-ALA. The cells were exposed to light from a bank of four fluorescent tubes (model 3026, Applied Photophysics, London, UK) emitting light mainly around 405 nm (Berg *et al.*, 1988). The fluence rate of the light reaching the cells was 36 W $m^{-2}$. The medium was removed immediately after illumination and replaced with 5-ALA-free RPMI-1640 containing 10% FCS.

### *Cell survival*

Cell survival was measured by the colony-forming test as previously described (Berg *et al.*, 1991). Approximately 500 cells were inoculated in 25 $cm^2$ plastic tissue culture flasks and treated with 5-ALA and light as described above. After photochemical treatment the V79 and WiDr cells were left for 5 and 10 days, respectively, at 37°C in serum-containing culture medium to allow for formation of countable colonies. The cells were then fixed in ethanol, stained with methylene blue and the colonies counted. The treatment of the cells with 5-ALA, as performed in the present study, did not reduce the colony-forming ability of the cells.

### *Measurements of cellular PpIX contents*

Approximately $5 \times 10^5$ WiDr and $4 \times 10^5$ V79 cells were seeded into 10 $cm^2$ dishes (Nunclon) and treated with 5-ALA as described above. The cells were then washed once in PBS and brought into a solution containing 1 M perchloric acid in 50% methanol by scraping with a Costar cell scraper. After 5 min of incubation the cell debris was removed by centrifugation. PpIX was quantitatively extracted from the cells with this procedure. The PpIX content of the samples was detected spectrofluorometrically using a Perkin Elmer LS50B spectrofluorometer. The PpIX was excited at 408 nm and the fluorescence was measured at 605 nm. A long pass cut-off filter (530 nm) was used on the emission side. A standard of known concentration was added to the samples at a concentration increasing the total fluorescence by 50–100%. The protein content was measured by the Lowry method (Lowry *et al.*, 1951).

### *High-performance liquid chromatography (HPLC)*

The porphyrins were extracted from the cells by scraping the cells in acidified methanol (5 $\mu l$ concentrated hydrochloric acid in 10 ml of methanol). The cell debris was pelleted and the supernatant collected. The porphyrins were concentrated by flushing the extracts with nitrogen until the volume was reduced to approximately 150–200 $\mu l$ and additionally precipitated proteins were pelleted. The supernatant (100 $\mu l$) was mixed with 235 $\mu l$ of 10 mM sodium phosphate, pH adjusted to approximately 10.5 by means of 5 M potassium hydroxide and directly used for HPLC analysis. The porphyrins were quantitatively extracted from the cells by this procedure.

The HPLC system consisted of a pump (Spectra Physics 8800), a reversed phase column [Supelcosil LC-18-T (4.6 × 250 mm), Supelco, Gland, Switzerland], an absorption detector (Spectra Physics Spectra 200) and an integrator (Spectra Physics Data-jet) connected to a computer. The start solvent was a mixture of methanol and water (70 : 30 by volume) containing 1.5 mM phosphate, adjusted to pH 7.0. The end solvent was a mixture of methanol and water (95 : 5 by volume) containing 1.5 mM phosphate, adjusted to pH 7.5. A 30 min linear gradient between 70% and 82% of methanol was applied followed by a 5 min linear gradient to 95% of methanol and 5 min at 95% of methanol. The absorption was detected at 405 nm.

### *Fluorescence microscopy*

Twenty-eight $cm^2$ dishes (Falcon 3002) were used in the microscopical studies. The cells were washed once with PBS and a cover glass was gently put on top of a PBS layer. The cells were subsequently studied by a Zeiss Axioplan microscope equipped with epifluorescence. A HBO/100 W mercury lamp was used for excitation. The cells and the cellular fluorescence were studied by means of a cooled charge-coupled device (CCD) camera (TE2, Astromed, Cambridge). A computer controlled the camera operation and was used for digital image processing and storage. The microscope was equipped with a 390–440 nm band pass excitation filter, a 470 nm dichroic beam splitter and a 600 nm long-pass filter.

### Results

### *Porphyrin synthesis in V79 and WiDr cells*

Porphyrin synthesis was stimulated by 5-ALA treatment both in the fast-growing V79 cells and in the more slowly growing WiDr cells (Figure 1). In both cell lines the same sigmoidal-shaped curves for the 5-ALA-dependent PpIX formation were found. The rate of PpIX synthesis reached in both cases a maximum, 280 ng PpIX (mg protein)$^{-1}$ for the WiDr cells and 55 ng PpIX (mg protein)$^{-1}$ for the V79 cells after 4 h of 5-ALA treatment. The maximum PpIX formation was achieved by treating the V79 cells with 0.1 mM 5-ALA, whereas 1.0 mM was necessary for reaching the maximum in WiDr cells. The colony-forming ability of both cell lines was inhibited by 4 h treatment with 5-ALA concentrations higher than 1.5 mM (data not shown).

### *Effect of iron chelators and DDC on PpIX accumulation*

Three different concentrations of 5-ALA were selected for studying the effect of iron chelators on PpIX accumulation, i.e. concentrations of 5-ALA inducing nearly no PpIX (0.025 mM and 0.1 mM 5-ALA for V79 and WiDr cells respectively), approximately 50% of maximum capacity for PpIX synthesis (0.05 mM and 0.3 mM 5-ALA for V79 and WiDr cells respectively), and maximum capacity for PpIX

synthesis (0.1 mM and 1 mM 5-ALA for V79 and WiDr cells respectively). In WiDr cells, EDTA treatments up to 100 $\mu$M induced only an insignificant increase in the biosynthesis of PpIX (Figure 2a). In Figure 3a the same results are plotted as the increase in PpIX accumulation relative to the accumulation in cells treated with 5-ALA only. As seen from the figure the relative effect of EDTA was most pronounced at the lowest concentrations of 5-ALA. A more substantial increase in PpIX accumulation was observed after cotreatment with DEF (Figure 2b). Similar to the effect of EDTA treatment, the most pronounced DEF-induced enhancement of PpIX accumulation was observed at the lowest concentration of 5-ALA applied (Figure 3b). At 0.1 mM 5-ALA, DEF (1000 $\mu$M) increased the PpIX accumulation more than 3-fold. At the same chelator concentration (100 $\mu$M), DEF still induced a more pronounced accumulation of PpIX than EDTA (Figure 3). In contrast to the iron chelators, the dihydropyridine DDC inhibited PpIX accumulation (Figure 2c), with the highest inhibition occurring at the lowest 5-ALA concentration applied (Figure 3c).

In V79 cells both EDTA and DEF significantly increased the accumulation of PpIX at all selected concentrations of 5-ALA (Figure 4). In particular, treatment with 1 mM DEF was highly efficient, and the accumulation of PpIX was enhanced 2.4-fold even at 0.1 mM 5-ALA. As in the case of WiDr cells, the relative chelator-induced increase in PpIX accumulation was inversely related to the applied concentration of 5-ALA (Figure 5). In V79 cells treated with 0.025 mM 5-ALA a more than 30-fold increase in PpIX accumulation was often observed by cotreatment with 1 mM DEF. To reveal the optimal 5-ALA concentration for DEF-induced enhancement of PpIX accumulation, the 5-ALA concentration was varied in the range 0–0.05 mM in the absence or presence of 1 mM DEF (Figure 6). DEF increased the PpIX accumulation in the whole range studied with a maximum enhancement at 0.020 mM 5-ALA. At the lowest concentrations of 5-ALA applied, the absolute relative increase varied between separate experiments as a result of a very low and

**Figure 1** PpIX formation in 5-ALA-treated V79 cells (●) and WiDr (○) cells. The cells were treated for 4 h in serum-free medium as described in Materials and methods. A close-up of the effect of treatment with less than 0.2 mM 5-ALA is shown in **b**. The curve for accumulation of PpIX in V79 cells is fitted to the following function: $y = 69.4$ than $(7848x^2 - 135x + 0.09)$. Bars (s.d.) are shown when larger than symbols.

**Figure 2** PpIX formation in WiDr cells treated with 5-ALA in combination with EDTA (**a**), DEF (**b**) or DDC (**c**). The cells were treated for 4 h in serum-free medium as described in Materials and methods with 5-ALA and 0 (○), 3 (▲), 10 (●), 30 (□), 100 (■) or 1000 (▽) $\mu$M EDTA, DEF or DDC. Bars (s.d.) are shown when larger than symbols.

**Figure 3** Relative increase in PpIX formation in WiDr cells treated with 5-ALA in combination with EDTA (**a**), DEF (**b**) or DDC (**c**). The PpIX formation is presented as relative to the amount of PpIX in cells treated with 5-ALA only. ▽, 0.1 mM 5-ALA; ●, 0.3 mM 5-ALA; ○, 1 mM 5-ALA. Bars (s.d.) are shown when larger than symbols.

**Figure 4** PpIX formation in V79 cells treated with 5-ALA alone (○) or in combination with 100 μM (●) or 1 mM (□) EDTA, 100 μM (■) or 1 mM (▽) DEF, or 100 μM (▲) DDC. The cells were treated for 4 h in serum-free medium as described in Materials and methods. Bars (s.d.) are shown when larger than symbols.

**Figure 5** Relative increase in PpIX formation in V79 cells treated with 5-ALA in combination with 100 μM (●) or 1 mM (□) EDTA, 100 μM (■) or 1 mM (▽) DEF, or 100 μM (▲) DDC. The PpIX formation is presented as relative to the amount of PpIX in cells treated with 5-ALA only. Bars (s.d.) are shown when larger than symbols.

variable formation of PpIX in cells treated in the absence of DEF. As in the case of WiDr cells, DDC substantially inhibited the accumulation of PpIX in the presence of 5-ALA (Figure 4). CaMgEDTA concentrations up to 1 mM induced an increase in PpIX accumulation which was lower than that of EDTA (data not shown).

The increased accumulation of PpIX caused by DEF in 5-ALA-treated cells can be visualised by subtraction of PpIX formed in the presence of 5-ALA only (Figure 7). In both cell lines the DEF-induced increase in PpIX accumulation seems to be saturable. When these results are presented in a double-reciprocal plot, the data fit well to linear regression curves (Figure 8). The dotted lines in Figure 7 are based on the linear regression curves obtained from Figure 8. The intercepts of the regression curves in Figure 8 with the ordinate axis is $[\Delta V_{max}]^{-1}$, i.e. the maximum effect of DEF on accumulation of PpIX at the selected concentration of 5-ALA. In both cell lines the same $\Delta V_{max}$ was obtained with the two highest concentrations of exogenously applied 5-ALA. These data indicate that these 5-ALA concentrations saturate the ferrochelatase with its substrate PpIX, and $V_{max}$ from these 5-ALA concentrations therefore reflect the maximal rate of iron insertion into PpIX in these cell lines under the present conditions. The $\Delta V_{max}$ after treatment with the lowest concentration of 5-ALA was substantially lower in both cell lines, indicating that at these concentrations 5-ALA induced formation of suboptimal concentrations of PpIX as a substrate for ferrochelatase. The 5-ALA-induced formation of PpIX and the maximum obtainable enhancement inducible by DEF is illustrated in Figure 9. As pointed out above, at the highest 5-ALA concentration the V79 cells and the WiDr cells form 50 and 280 ng PpIX (mg protein)$^{-1}$ after 4 h of incubation respectively. The maximum DEF-inducible enhancement of PpIX accumulation is almost similar in V79 and WiDr cells, i.e. 93 and 124 ng PpIX (mg protein $\times$ 4 h)$^{-1}$ respectively (Figure 9a). Thus, the maximal accumulation of

**Figure 6** Absolute (○,●) and relative (□) formation of PpIX in V79 cells treated with 5-ALA in the absence (○) or presence (●) of 1 mM DEF. The cells were treated for 4 h in serum-free medium as described in Materials and methods.

**Figure 7** DEF-induced increase in PpIX accumulation in 5-ALA-treated WiDr (**a**) and V79 (**b**) cells. The cells were treated for 4 h in serum-free medium in the presence of 0.1 mM (WiDr) or 0.025 mM (V79) (□), 0.3 mM (WiDr) or 0.05 mM (V79) (●), 1 mM (WiDr) or 0.1 mM (V79) (○) 5-ALA and DEF as indicated. The increase in PpIX accumulation is deduced by subtraction of PpIX in 5-ALA-treated cells from PpIX in cells treated with the same concentration of 5-ALA and DEF.

**Figure 8** Double-reciprocal plots of the DEF-induced increase in PpIX accumulation in 5-ALA-treated WiDr (**a**) and V79 (**b**) cells. The cells were treated for 4 h in serum-free medium in the presence of 0.1 mM (WiDr) or 0.025 mM (V79) (□), 0.3 mM (WiDr) or 0.05 mM (V79) (●), 1 mM (WiDr) or 0.1 mM (V79) (○) 5-ALA and DEF as indicated. The increase in PpIX accumulation is deduced by subtraction of PpIX in 5-ALA-treated cells from PpIX in cells treated with the same concentration of 5-ALA and DEF.

PpIX in the absence of iron available for the ferrochelatase is 140 and 404 ng PpIX (mg protein $\times 4\,h)^{-1}$ for V79 and WiDr cells respectively. At these concentrations of 5-ALA, DEF can maximally increase the accumulation of PpIX 3- and 1.4-fold in V79 and WiDr cells respectively. In a similar way it can be estimated that for cells treated with the lowest concentrations of 5-ALA used in this study, DEF may enhance PpIX accumulation 44- and 3.5-fold in V79 and WiDr cells respectively (Figure 9).

### *Analysis of accumulated porphyrin intermediates*

The fluorescence emission spectrum of PpIX is slightly red-shifted compared with the two other main intermediates in the porphyrin synthesis pathway, uro- and coproporphyrin (Granick *et al.*, 1975). In our analysis of cellular contents of porphyrins, the fluorescence emission spectra were similar to that of PpIX, indicating that mainly PpIX was formed after all types of treatments performed. This was confirmed by HPLC analysis of extracts from cells treated with 5-ALA in the presence or absence of modulators of the haem synthesis pathway (Figure 10). No intermediate other than PpIX was observed. These analyses clearly show that neither the iron chelators, DEF or EDTA, nor DDC in combination with 5-

**Figure 9** Summary of the optimal DEF-inducible effect on PpIX accumulation in WiDr and V79 cells cotreated with 5-ALA as indicated in the figure. □, PpIX in cells treated with 5-ALA only; ▨, maximal DEF-induced increase in PpIX accumulation in 5-ALA-treated cells (deduced from intercepts with the ordinates in Figure 8); ■, total accumulation of PpIX in cells treated with 5-ALA under conditions where ferrochelatase activity is maximally inhibited by DEF, i.e. the sum of □ and ▨. The maximal DEF-induced increase in PpIX accumulation is deduced from Figure 8.

ALA treatment induce accumulation of other porphyrin intermediates than does 5-ALA alone.

### *Intracellular localisation of PpIX*

The intracellular localisation of PpIX in cells treated with 5-ALA in the presence or absence of iron chelators was assessed by fluorescence microscopy (Figure 11). PpIX seems from these studies to localise in two or more compartments. A large fraction of PpIX is diffusely located all over the cells including the outer lining of the cells. This indicates that a fraction of PpIX is associated with the plasma membrane. The strong fluorescence at the border between the cells may support this conclusion, but may also be due to PpIX located in the extracellular space between interdigitating membranes of the cells. Additionally, the low fluorescence intensity from the nuclear area indicate cytoplasmic, most likely membrane-associated, localisation of PpIX. As seen in Figure 11 this cytoplasmically located PpIX is to a large extent focused in a perinuclear spot. No difference in the distribution of PpIX was observed between cells treated with 5-ALA alone or in combination with iron chelators (Figure 11).

### *5-ALA-PCT in combination with iron chelators and DDC*

EDTA, DEF and DDC in the absence or presence of 5-ALA did not induce any cytotoxic effects during the experiments as revealed by the trypan blue exclusion method. However, to reveal any long-term effects of the drugs, a clonogenicity test

**Figure 10** HPLC analysis of porphyrin extracts from V79 cells treated with 5-ALA alone or in combination with the compounds described on the figure. The cells were treated for 4 h in serum-free medium and porphyrins extracted from the cells as described in Materials and methods. Standards for uroporphyrin (URO), coproporphyrin (COPRO) and PpIX are included.

was performed on the cells. Only DEF induced a substantial inhibition of the colony-forming ability of the cells and only at concentrations higher than 0.1 mM (data not shown). EDTA was found to reduce the number of colonies in some cases. This was assumed to be due to the effect of EDTA on the binding of cells to the substratum.

The cytotoxic effect of DEF prohibited comparative studies of the combination of 5-ALA-PCT and optimal concentrations of iron chelators. Therefore, suboptimal concentrations (100 μM) of the modulators have been combined with 5-ALA-PCT in a colony-forming assay. The sensitivity of the cells to photoinactivation was substantially enhanced by the iron chelators only at the low concentration of 5-ALA (Figure 12). Also in accordance with the results on PpIX accumulation, DEF sensitised the cells to photoinactivation to a higher extent than EDTA. DDC did not significantly change the sensitivity of the cells to ALA-PCT despite a reduced formation of PpIX in the presence of DDC.

### Discussion

The purpose of the current studies has been to elucidate further the influence of iron chelators on the rate of PpIX accumulation. This has been performed by studying the effect of the iron chelators, EDTA and DEF, at different concentrations of chelators and 5-ALA. Both chelators have previously been applied to cells *in vitro* and shown to increase the accumulation of PpIX and the sensitivity of the cells to photoinactivation in the presence of 5-ALA (Hanania and Malik, 1992; Ortel *et al.*, 1993). However, a complete study to reveal the maximal possible gain in using iron chelators at different concentrations of 5-ALA has not previously been performed. The two iron chelators compared in this study have different physicochemical properties. EDTA is a membrane-impermeable chelator (Richardson *et al.*, 1994) with affinity for several cations, of which that for $Fe^{3+}$ is notably high ($logK_m \approx 25$) compared with that for, for example, $Ca^{2+}$ ($logK_m \approx 10.7$; Keberle, 1964). EDTA has been used clinically in a cream together with 5-ALA and dimethyl sulphoxide (DMSO) for topical application (Heyerdahl *et al.*, 1993; Orenstein *et al.*, 1994). The sideramine DEF, on the other hand, is a highly specific membrane-permeable iron chelator with very high affinity for iron ($logK = 31$; Keberle, 1964). DEF has so far not been used in clinical 5-ALA-PCT, but is promising as an anti-

**Figure 11** Fluorescence micrographs of V79 cells treated with 0.1 mM 5-ALA alone or in combination with iron chelators as indicated on the figure. The cells were treated for 4 h in serum-free medium as described in Materials and methods.

neoplastic agent alone and is currently used clinically to treat iron overload diseases such as thalassemia and transfusion-related overload (Blatt, 1994; Richardson *et al.*, 1994). In the present study both chelators were found to increase the accumulation of PpIX in 5-ALA-treated cells, although DEF was more efficient on a molar basis (Figures 3 and 5). This is most likely because of the intracellular localisation of DEF, the slow release of iron from cells (Richardson *et al.*, 1994) and EDTA's binding to other cations. DEF supposedly binds iron from the intracellular iron pool which most likely consists of small molecular weight iron chelates of sugars, amino acids and nucleotides, all with low affinity for iron (Jacobs, 1977) in addition to ferritin. In this way DEF may reduce the amount of iron accessible for the ferrochelatase. On the basis of the present results, it may be advantagous to use DEF instead of EDTA as enhancer of PpIX accumulation. Our recent results also indicate that the gain in using DEF is significantly higher than that in using EDTA in combination with 5-ALA for the formation of fluorescing porphyrins in mouse skin (Peng *et al.*, 1996).

The iron chelator-induced increase in PpIX accumulation ($\Delta V$) was found to be saturable (Figure 7). The linear relationship between $\Delta V^{-1}$ and $[DEF]^{-1}$ indicates that the rate of iron incorporation into PpIX in cells follows Michaelis–Menten kinetics. This is in accordance with data on purified ferrochelatase (Taketani and Tokunaga, 1982; Ferreira, 1994). In both V79 and WiDr cells the accumulation of PpIX is increased from the medium to the highest concentrations of 5-ALA (Figure 1), whereas the double-reciprocal curves for DEF-induced increase in PpIX accumulation (Figure 8) have the same intercepts with the ordinate for these concentrations of 5-ALA. This indicates that saturating concentrations of PpIX are provided to the ferrochelatase when WiDr and V79 cells are treated with 5-ALA concentrations above 0.3 mM and 0.05 mM respectively. Furthermore, assuming the mean diameter of WiDr and V79 cells to be 15 and 11 $\mu$m, respectively, the mean maximal PpIX concentration in both cell lines will be approximately 50–150 $\mu$M after 4 h of 5-ALA treatment. This is similar to the $K_{m,PpIX}$ of isolated ferrochelatase (50–100 $\mu$M; Taketani

**Figure 12** Surviving fraction of WiDr and V79 cells treated with 5-ALA (at concentrations as indicated on the figure) alone (▭) or in combination with 100 $\mu$M EDTA (▨), 100 $\mu$M DEF (■) or 100 $\mu$M DDC (▤). Light doses were adjusted so that 30–50% of the cells treated with 5-ALA alone were killed. The cells were exposed to the following doses of light: V79 cells, 0.025 mM 5-ALA: 15 min; 0.1 mM 5-ALA: 2 min. WiDr cells, 0.1 mM 5-ALA: 4 min; 1 mM 5-ALA: 0.5 min. Survival was measured in triplicate by a colony-forming assay as described in Materials and methods.

and Tokunaga, 1982; Ferreira, 1994). However, PpIX is clearly not homogeneously distributed intracellularly but highly concentrated in mitochondria (Iinuma *et al.*, 1994), lysosomes (Gaullier *et al.*, 1995) and the plasma membrane (Figure 11) (Gaullier *et al.*, 1995), which may also suggest saturating PpIX concentrations around the mitochondrial ferrochelatase. Hence, the present results indicate that the intercept with the ordinate in the double-reciprocal plot can be used to estimate ferrochelatase activity in cells. However, it should be emphasised that the values obtained by this method may differ from the maximum ferrochelatase activity, since availability to iron may be rate limiting even in the absence of iron chelators. Furthermore, 5-ALA- and DEF-induced changes in the intracellular concentrations of modulators of ferrochelatase activity may influence the estimated activities. For example, haemin, the oxidised form of haem, is known to inhibit ferrochelatase activity (Rossi *et al.*, 1990). It cannot be excluded that DEF may be an inducer of other enzymes in the pathway leading to a haem formation (Sassa and Bernstein, 1977).

There are several examples from the literature suggesting that the ferrochelatase activity is reduced in neoplastically transformed tumours compared with their normal counterparts (Rebeiz *et al.*, 1992; Dailey and Smith, 1984; van Hillegersberg *et al.*, 1992). The present results indicate that in WiDr cells there is a 3-fold higher capacity for formation of PpIX than the capacity for incorporation of ferrous iron into PpIX (Figure 9). On the other hand, in V79 cells the same ratio is only 1.5 : 1. This difference between the two cell lines may be owing to the difference in origin of these cell lines. V79 cells are derived from normal Chinese hamster lung fibroblasts, while WiDr cells are derived from a human adenocarcinoma. Thus, the V79 cells may, to some extent, resemble non-transformed cells with respect to the biosynthetic properties. It would be of great interest to perform studies similar to the present ones on normal cells and their neoplastic counterparts. One may speculate that the enhancement of PpIX accumulation by the use of iron chelators in combination with 5-ALA may be more pronounced in normal cells than in neoplastically transformed cells. Thus, systemic treatment with iron chelators in combination with 5-ALA may reduce the selectivity of the treatment. On topical application there may be a therapeutic gain in the deeper layers of the tumour where less PpIX is formed, in using a combination of 5-ALA and iron chelators (Orenstein *et al.*, 1994). As seen in the present study, the largest benefit of co-treatment with iron chelators is found at low concentrations of 5-ALA (Figures 3 and 5).

The iron chelator-induced accumulation of PpIX was dependent upon the cell line and the concentration of 5-ALA (Figure 9). The highest effect was observed at the lowest concentration of 5-ALA, and the accumulation could be increased to a higher extent in V79 cells than in WiDr cells. At low concentrations of 5-ALA (<0.025 mM) virtually no PpIX is accumulated in V79 cells. At these low concentrations of 5-ALA ferrochelatase is most likely able to convert all PpIX formed into haem. This is supported by the nearly linear relationship between PpIX accumulation and 5-ALA concentration when the ferrochelatase activity is inhibited by DEF treatment (Figure 6).

The iron chelators were also found to enhance the sensitivity of the cells to photoinactivation (Figure 12). The studies of PpIX accumulation in cells indicated that iron chelators should be more efficient in increasing the sensitivity of the cells to photoinactivation at low than at high concentrations of 5-ALA. Furthermore, DEF should be expected to be more efficient than EDTA in increasing the sensitivity of the cells to photoinactivation. This was indeed found to be the case for both V79 and WiDr cells (Figure 12). The enhancement was found to be strictly dependent on the amount of PpIX in the cells (data not shown), indicating that the iron chelators do not change the intracellular localisation of PpIX or in other ways change the quantum yield for photoinactivation of the accumulated porphyrins. The fluorescence micrography studies confirmed that no major changes in intracellular localisation of PpIX was induced by the modulators (Figure 11). HPLC analysis showed that only PpIX was accumulated in the presence or absence of iron chelators in 5-ALA-treated cells (Figure 10).

DDC is well known to elevate the haem precursor level in mice and rats (Ortiz de Montellano *et al.*, 1981) by markedly decreasing the hepatic ferrochelatase (Brady and Lock, 1992;

Kimmett *et al.*, 1992). This is not due to a direct effect of DDC, but rather to the accumulation of N-methyl protoporphyrin IX in hepatocytes by transfer of methyl groups from DDC to PpIX (Ortiz de Montellano *et al.*, 1981). *N*-methyl protoporphyrin IX is a potent inhibitor of ferrochelatase (Kimmett *et al.*, 1992). The present results indicate that the transfer of the methyl group from DDC to PpIX does not occur in non-hepatic cells. In contrast, it was found that the accumulation of PpIX was reduced by DDC treatment of cells exposed to 5-ALA (Figures 2–5). This inhibition may be cell line dependent (Schoenfeld *et al.*, 1994). Since no porphyrins other than PpIX were found in 5-ALA- and DDC-treated cells (Figure 10), DDC is likely to interact at a step in the haem biosynthetic pathway before the formation of uroporphyrin or with the uptake of 5-ALA into the cells.

**Acknowledgement**
This work was supported by the Norwegian Cancer Society and the Research Council of Norway.

### References

AULER H AND BANZER G. (1943). Untersuchungen uber die rolle der porphyrine bei geschwulstkranken menschen und tieren. *Z Krebsforschungen*, **53**, 65–68.

BERG K, HOVIG E AND MOAN J. (1988). Sister chromatid exchanges induced by photodynamic treatment of cells in the presence of Photofrin II, aluminum phthalocyanine tetrasulfonate and tetra(3-hydroxyphenyl)porphyrin. In *Light in Biology and Medicine*. Douglas J, Dall'Aqua F, Moan J. (eds) pp. 95–103. Plenum Press: New York.

BERG K, MADSLIEN K, BOMMER JC, OFTEBRO R, WINKELMAN JW AND MOAN J. (1991). Light induced relocalization of sulfonated meso-tetraphenylporphines in NHIK 3025 cells and effects of dose fractionation. *Photochem. Photobiol.*, **53**, 203–210.

BLATT J. (1994). Deferoxamine in children with recurrent neuroblastoma. *Anticancer Res.*, **14**, 2109–2112.

BRADY AM AND LOCK EA. (1992). Inhibition of ferrochelatase and accumulation of porphyrins in mouse hepatocyte cultures exposed to porphyrinogenic chemicals. *Arch. Toxicol.*, **66**, 175–181.

DAILEY HA AND SMITH A. (1984). Differential interaction of porphyrins used in photoradiation therapy with ferrochelatase. *Biochem. J.*, **223**, 441–445.

EL-SHARABASY MM, EL-WASEEF AM, HAFEZ MM AND SALIM SA. (1992). Porphyrin metabolism in some malignant diseases. *Br. J. Cancer*, **65**, 409–412.

FERREIRA GC. (1994). Mammalian ferrochelatase. Overexpression in *Escherichia coli* as a soluble protein, purification and characterization. *J. Biol. Chem.*, **269**, 4396–4400.

GAULLIER JM, GEZE M, SANTUS R, SA E, MELO T, MAZIERE JC, BAZIN M, MORLIERE P AND DUBERTRET L. (1995). Subcellular localization of and photosensitization by protoporphyrin IX in human keratinocytes and fibroblasts cultivated with 5-aminolevulinic acid. *Photochem. Photobiol.*, **62**, 114–122.

GRANICK S, SINCLAIR P, SASSA S AND GRIENINGER G. (1975). Effects by heme, insulin, and serum albumin on heme and protein synthesis in chick embryo liver cells cultured in a chemically defined medium, and a spectrofluorometric assay for porphyrin composition. *J. Biol. Chem.*, **250**, 9215–9225.

HANANIA J AND MALIK Z. (1992). The effect of EDTA and serum on endogenous porphyrin accumulation and photodynamic sensitization of human K562 leukemic cells. *Cancer Lett.*, **65**, 127–131.

HENDERSON B AND DOUGHERTY TJ. (1992). How does photodynamic therapy work? *Photochem. Photobiol.*, **55**, 145–157.

HEYERDAHL H, WARLOE T, PENG Q, SVANBERG K, MOAN J, STEEN H, SVAASAND L AND GIERCKSKY K. (1993). Dosimetry and light distribution systems for photodynamic therapy at the Norwegian Radium Hospital. In *Photodynamic Therapy of Cancer*, Jori G, Moan J, Star W. (eds) pp. 27–36. SPIE: Bellingham.

IINUMA S, FARSHI SS, ORTEL B AND HASAN T. (1994). A mechanistic study of cellular photodestruction with 5-aminolaevulinic acid-induced porphyrin. *Br. J. Cancer*, **70**, 21–28.

JACOBS A. (1977). Low molecular weight intracellular iron transport compounds (review). *Blood*, **50**, 433–439.

KEBERLE H. (1964). The biochemistry of desferrioxamine and its relation to iron metabolism. *Anns. N. Y. Acad. Sci.*, **19**, 758–768.

KELLY JF AND SNELL ME. (1976). Hematoporphyrin derivative: a possible aid in the diagnosis and therapy of carcinoma of the bladder. *J. Urol.*, **115**, 150–157.

KENNEDY JC, POTTIER RH AND PROSS DC. (1990). Photodynamic therapy with endogenous protoporphyrin IX: basic principles and present clinical experience. *J. Photochem. Photobiol. B Biol.*, **6**, 143–148.

KENNEDY JC AND POTTIER RH. (1992). Endogenous protoporphyrin IX, a clinically useful photosensitizer for photodynamic therapy (review). *J. Photochem. Photobiol. B Biol.*, **14**, 275–292.

KIMMETT SM, WHITNEY RA AND MARKS GS. (1992). Evidence for the stereoselective inhibition of chick embryo hepatic ferrochelatase by N-alkylated porphyrins. II. *Mol. Pharmacol.*, **42**, 307–310.

LIPSON RL, BALDES EJ AND GRAY M. (1967). Hematoporphyrin derivative for detection and management of cancer. *Cancer*, **20**, 2255–2257.

LOWRY OH, ROSEFROUGH NJ, FARR AL AND RANDALL RJ. (1951). Protein measurement with the Folin phenol reagent. *J Biol. Chem.*, **193**, 265–275.

MALIK Z AND LUGACI H. (1987). Destruction of erythroleukaemic cells by photoactivation of endogenous porphyrins. *Br. J. Cancer*, **56**, 589–595.

MOAN J AND BERG K. (1992). Photochemotherapy of cancer: experimental research (review). *Photochem. Photobiol.*, **55**, 931–948.

NOGUCHI P, WALLACE R, JOHNSON J, EARLEY EM, O'BRIEN S, FERRONE S, PELLEGRINO MA, MILSTEIN J, NEEDY C, BROWNE W AND PETRICCIANI J. (1979). Characterization of the WIDR: a human colon carcinoma cell line. *In Vitro*, **15**, 401–408.

ORENSTEIN A, KOSTENICH G, TSUR H, ROITMAN L, EHRENBERG B AND MALIK Z. (1994). Photodynamic therapy of human skin tumors using topical application of 5-aminolevulinic acid, DMSO and EDTA. In *Photodynamic Therapy of Cancer II*. Brault D, Jori G, Moan J, Ehrenberg B. (eds) pp. 100–105. SPIE: Bellingham.

ORTEL B, TANEW A AND HONIGSMANN H. (1993). Lethal photosensitization by endogenous porphyrins of PAM cells – modification by desferrioxamine. *J. Photochem. Photobiol. B Biol.*, **17**, 273–278.

ORTIZ DE MONTELLANO PR, BEILAN HS AND KUNZE KL. (1981). N-Alkylprotoporphyrin IX formation in 3,5-dicarbethoxy-1,4-dihydrocollidine-treated rats. Transfer of the alkyl group from the substrate to the porphyrin. *J. Biol. Chem.*, **256**, 6708–6713.

PENG Q, MOAN J, IANI V AND NESLAND JM. (1996). Effect of desferrioxamine on production of ALA-induced protoporphyrin IX in normal mouse skin. In *Photochemotherapy:Photodynamic Therapy and Other Modalities*. Eherenberg B, Jori G, Moan J. (eds) pp. 51–57. SPIE:Bellingham.

POLICARD A. (1924). Etudes sur les aspects offert par des tumeurs experimentales examinees a la lumiere de Wood. *C R Soc. Biol.*, **91**, 1423–1424.

POPE AJ, MACROBERT AJ, PHILLIPS D AND BOWN SG. (1991). The detection of phthalocyanine fluorescence in normal rat bladder wall using sensitive digital imaging microscopy. *B. J. Cancer*, **64**, 875–879.

REBEIZ N, REBEIZ CC, ARKINS S, KELLEY KW AND REBEIZ CA. (1992). Photodestruction of tumor cells by induction of endogenous accumulation of protoporphyrin IX: enhancement by 1,10-phenanthroline. *Photochem. Photobiol.*, **55**, 431–435.

RICHARDSON D, PONKA P AND BAKER E. (1994). The effect of the iron(III) chelator, desferrioxamine, on iron and transferrin uptake by the human malignant melanoma cell. *Cancer Res.*, **54**, 685–689.

RITTENHOUSE-DIAKUN K, VAN LEENGOED H, MORGAN J, HRYHORENKO E, PASZKIEWICZ G, WHITAKER JE AND OSEROFF AR. (1995). The role of transferrin receptor (CD71) in photodynamic therapy of activated and malignant lymphocytes using the heme precursor delta-aminolevulinic acid (ALA). *Photochem. Photobiol.*, **61**, 523–528.

ROSSI E, ATTWOOD PV, GARCIA-WEBB P AND COSTIN KA. (1990). Inhibition of human lymphocyte ferrochelatase activity by hemin. *Biochim. Biophys. Acta*, **1038**, 375–381.

SASSA S AND BERNSTEIN SE. (1977). Levels of delta-aminolevulinate dehydratase, uroporphyrinogen-I synthase, and protoporphyrin IX in erythrocytes from anemic mutant mice. *Proc. Natl Acad. Sci. USA*, **74**, 1181–1184.

SCHOENFELD N, EPSTEIN O, LAHAV M, MAMET R, SHAKLAI M AND ATSMON A. (1988). The heme biosynthetic pathway in lymphocytes of patients with malignant lymphoproliferative disorders. *Cancer Lett.*, **43**, 43–48.

SCHOENFELD N, MAMET R, NORDENBERG Y, SHAFRAN M, BABUSHKIN T AND MALIK Z. (1994). Protoporphyrin biosynthesis in melanoma B16 cells stimulated by 5-aminolevulinic acid and chemical inducers: characterization of photodynamic inactivation. *Int. J. Cancer*, **56**, 106–112.

TAKETANI S AND TOKUNAGA R. (1982). Purification and substrate specificity of bovine liver-ferrochelatase. *Eur. J. Biochem.*, **127**, 443–447.

VAN HILLEGERSBERT R, VAN DEN BERT JW, KORT WJ, TERPSTRA OT AND WILSON JH. (1992). Selective accumulation of endogenously produced porphyrins in a liver metastasis model in rats. *Gastroenterology*, **103**, 647–651.