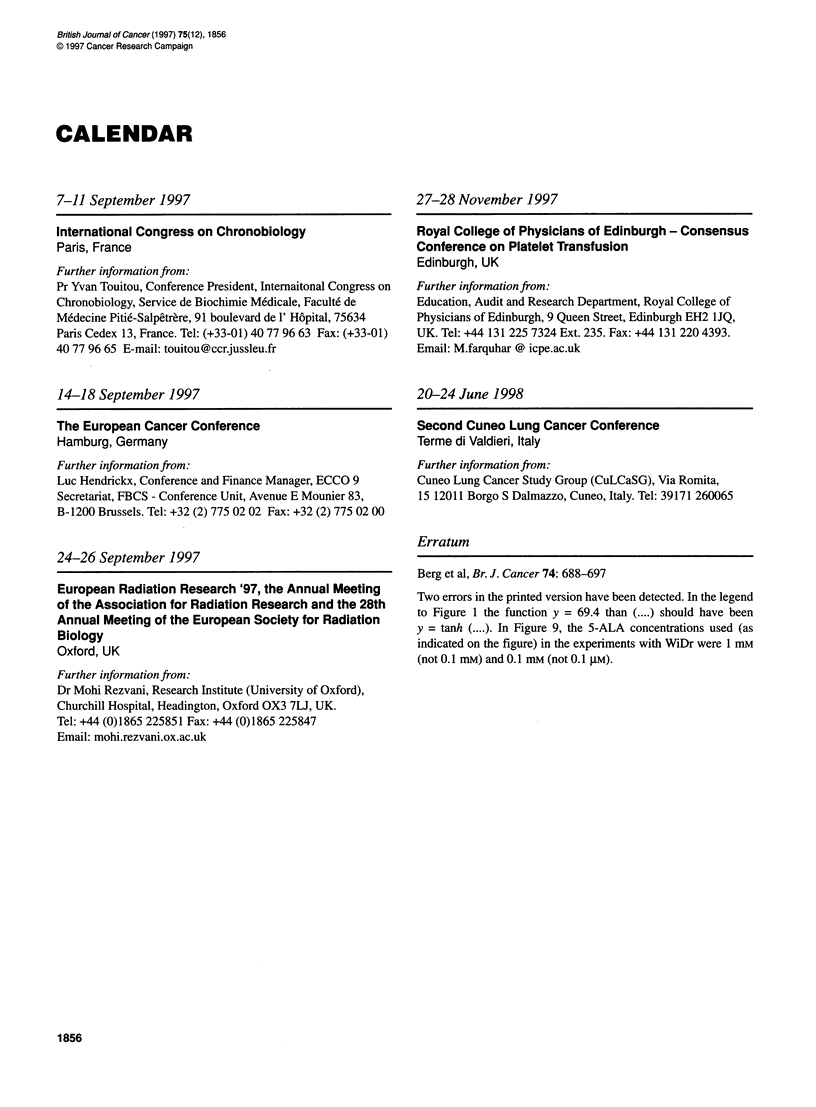# Erratum

**Published:** 1997

**Authors:** 


### *Erratum*

Berg et al, *Br. J. Cancer* **74**: 688–697

Two errors in the printed version have been detected. In the legend to Figure 1 the function $y$ = 69.4 than (....) should have been $y$ = tan$h$ (....). In Figure 9, the 5-ALA concentrations used (as indicated on the figure) in the experiments with WiDr were 1 mM (not 0.1 mM) and 0.1 mM (not 0.1 μM).